# Acidic Digestion in a Teleost: Postprandial and Circadian Pattern of Gastric pH, Pepsin Activity, and Pepsinogen and Proton Pump mRNAs Expression

**DOI:** 10.1371/journal.pone.0033687

**Published:** 2012-03-20

**Authors:** Manuel Yúfera, Francisco J. Moyano, Antonio Astola, Pedro Pousão-Ferreira, Gonzalo Martínez-Rodríguez

**Affiliations:** 1 Instituto de Ciencias Marinas de Andalucía (ICMAN-CSIC). Apartado Oficial, Puerto Real, Cadiz, Spain; 2 Department of Applied Biology, EPSs, University of Almería, Almería, Spain; 3 Department of Biomedicine and Biotechnology, Faculty of Sciences, University of Cádiz, Puerto Real, Spain; 4 INRB, I.P.- IPIMAR Av, 5 de Outubro s/n., Olhão, Portugal; University of Chicago, United States of America

## Abstract

Two different modes for regulation of stomach acid secretion have been described in vertebrates. Some species exhibit a continuous acid secretion maintaining a low gastric pH during fasting. Others, as some teleosts, maintain a neutral gastric pH during fasting while the hydrochloric acid is released only after the ingestion of a meal. Those different patterns seem to be closely related to specific feeding habits. However, our recent observations suggest that this acidification pattern could be modified by changes in daily feeding frequency and time schedule. The aim of this study was to advance in understanding the regulation mechanisms of stomach digestion and pattern of acid secretion in teleost fish. We have examined the postprandial pattern of gastric pH, pepsin activity, and mRNA expression for pepsinogen and proton pump in white seabream juveniles maintained under a light/dark 12/12 hours cycle and receiving only one morning meal. The pepsin activity was analyzed according to the standard protocol buffering at pH 2 and using the actual pH measured in the stomach. The results show how the enzyme precursor is permanently available while the hydrochloric acid, which activates the zymogen fraction, is secreted just after the ingestion of food. Results also reveal that analytical protocol at pH 2 notably overestimates true pepsin activity in fish stomach. The expression of the mRNA encoding pepsinogen and proton pump exhibited almost parallel patterns, with notable increases during the darkness period and sharp decreases just before the morning meal. These results indicate that white seabream uses the resting hours for recovering the mRNA stock that will be quickly used during the feeding process. Our data clearly shows that both daily illumination pattern and feeding time are involved at different level in the regulation of the secretion of digestive juices.

## Introduction

Two basic types of digestive tract can be observed in the different fish species, with and without stomach. In species with a stomach, the adult-mode of food processing implies an acid digestion phase and consequently a highly efficient extracellular digestion of proteins [1], [2]. In these species, the development of the stomach generally starts several days or weeks after the onset of exogenous feeding and it is widely accepted that the juvenile stage is attained once the stomach is morphologically completed and becomes fully functional, acting both as a reaction chamber and a food reservoir. This step marks the definitive change in feeding patterns, from a relatively continuous foraging on planktonic prey to a wide range of different species-specific feeding habits. In juvenile and adult fish, the gastric glands covering the inner layer of the stomach follow a species-specific pattern and produce both pepsinogen and proton pump H^+^/K^+^-ATPase. This enzyme is responsible for the secretion of the hydrochloric acid that decreases gastric pH and induces the conversion of pepsinogen into pepsin. The corresponding genes codifying these enzymes are also expressed in the gastric glands [3], [4], [5], [6].

Two different modes for regulation of stomach acid secretion have been described in vertebrates. One mode, characterized by a continuous acid secretion and the maintenance of a low pH during fasting has been described in humans [7], [8], dogs [9], some elasmobranchids [10] and some teleost fish [11], [12], [13]. In contrast, a mode characterized by maintenance of a neutral gastric pH during fasting, on which hydrochloric acid is released only after the ingestion of a meal, has been reported in some snakes [14], [15] and other teleosts [16], [17], [18]. Those different patterns seem to be closely related to specific feeding habits. While frequent feeders, as well as species faced to unpredictable food availability, tend to maintain a low gastric pH [10], [19], less frequent feeders may recover a neutral gastric pH between meals [15], [20]. However, a recent study [21] suggests that, at least in some species, gastric acidification could be modified by changes in daily feeding patterns and feeding frequency. This adaptability in the digestive function may be related to the ability to anticipate some physiological responses exhibited by many vertebrates in order to optimize the digestion process.

Feeding activity in most fish is related both to circadian and yearly seasonal cycles conditioning availability and composition of their prey. Therefore, wild fish must cope with a wide range of different feeding conditions, changing from several meals per day to long fasting periods. A better understanding of the action mechanisms, which ensure the optimum physiological response under such a variety of feeding situations, may result in interesting applications for cultured fish, but this requires getting a better knowledge on the basic response to a single meal. The present study was designed to get an insight into the daily regulation of stomach digestion and pattern of acid secretion in teleost fish. The selected species was the white seabream, *Diplodus sargus*, a sparid inhabiting the Eastern Atlantic coast (from the Bay of Biscay to South Africa) and the Meditteranean Sea. This species has a high commercial importance and a great interest in the Mediterranean aquaculture due to the reported decrease in its wild populations [22]. The white seabream is a generalist feeder eating macroalgae, invertebrates (crustaceans, gastropods, bivalves, echinoderms and polychetes) and fish [23], [24], [25]. In addition, as most sparids, white seabream is a visual feeder eating preferentially during the daytime.

A preliminary characterization of the development of gastric acidification was carried out from early to adult stages to ensure that the selected experimental fish possessed a fully developed acid digestion. After providing a single meal to juvenile fish, changes in gastric pH and pepsin activity, as well as in mRNA expression of their corresponding precursors (proton pump and pepsinogen) were assessed during a complete daily cycle. Assays of pepsin activity are usually carried out at pH 2 [26] since maximum activity of the enzyme in different vertebrates has been detected within a pH range between 1.5 and 3.5 [27], [28], [29], [30], [31], [32], [33]. Nevertheless, such a low value is only reached occasionally and during a short while in the stomach of most teleost fish and, as suggested by Cox and Secor [15], measures done at such low pH should reflect more the presence of pepsinogen than of active pepsin. In the present study a more realistic estimation of pepsin activity was obtained through assays carried out at the actual pH measured in the stomach of each sampled fish.

## Materials and Methods

### Fish maintenance and experimental conditions

All experimental procedures complied with the Guidelines of the European Union Council (86/609/EU) for the use and experimentation of laboratory animals and was reviewed and approved by the Spanish National Research Council (CSIC) bioethical committee.

White seabream (*Diplodus sargus*) eggs were obtained at the IPIMAR hatchery facilities (Portugal) and transported to the rearing facilities of the Instituto de Ciencias Marinas de Andalucía (Spain). Larvae were reared in 300-L tanks at a temperature of 19.5±1°C and a salinity of 33 g L^−1^. Larvae were fed rotifers (*Brachionus plicatilis*) and microalgae (*Nannochloropsis gaditana* and *Isochrysis galbana* T-ISO) from first-feeding to 18 days after hatching (DAH), *Artemia* nauplii and *Artemia* metanauplii enriched with *I. galbana* (T-ISO) from 12 DAH onwards. The weaning onto commercial feeds was done between 35–50 DAH.

For the characterization of the acidification capacity from larvae to adult, the gastric pH was determined in individuals born from different egg batches with a wet weight ranging between 30 mg and 470 g. Measurements were done at 12:00 h, three hours after the morning food supply to standardise feeding related pH changes.

For the post-prandial study, juveniles with a wet weight ranging between 1.0 and 2.5 g were kept in 300-L tanks at 20°C 33 g L^−1^ salinity and 12L/12D photoperiod and adapted to the experimental conditions and feeding schedule during 1 month. The experiment was initiated by feeding the fish with a single morning meal (commercial dry feed) at 9:00 and removing uneaten food was 2 hours later to prevent further ingestion. From this moment on the fish were regularly sampled during a 24 h period. The first sample was taken just after the food supply and the last one just after the start of the light period and before the next food supply.

### Gastric pH and pepsin activity measurements

For pH determinations, the fish were anaesthetised with ethyl-4-aminobenzoate and then dissected to make the digestive tract accessible. Measurements were taken in still living animals using a pH microelectrode (WPI, Minicombo PH660) [17], [34]. The tip of the microelectrode (diameter 660 µm) was inserted in small slits made in the stomach. The measurements were done in the cardiac section with the tip of the electrode touching the mucosa of the stomach. The stomach was then removed and immediately frozen at −20°C for the biochemical analysis of pepsin or immersed in RNA-later for the molecular analysis. Stomach repletion was estimated as the mean of the different analysed individuals, considering four subjective levels (0, 33, 66 and 100%) ranging from empty to completely full.

Pepsin activity was analysed using the method detailed by Anson [26] modified by Díaz et al. [35] using hemoglobin as substrate, being one unit of activity defined as 1 µg of tyrosine released per minute. Assays were carried out both at pH 2 and at the specific pH determined in each sampling point. The activity was expressed in relation to soluble protein in extracts (specific activity).

### Pepsinogen and proton pump cDNA cloning

*D. sargus* pepsinogen and proton pump cDNAs were cloned using RT-PCR and library screening strategies. Total RNA from a section of adult stomach was extracted using the RNeasy mini kit with the on column DNase-free RNase digestion for the removal of genomic DNA contamination (Qiagen). 2 µg of total RNA was reverse transcribed into cDNA using the RETROscript kit (Ambion). The product was amplified in a thermocycler Gene Amp PCR system 2700 (Applied Biosystems, Life Technologies), using Platinum®Taq DNA polymerase (Invitrogen, LifeTechnologies) and primers for pepsinogen and proton pump (Table 1) from winter flounder [36], in a total volume of 25 µl under the next conditions: 2 min at 94°C; 32 cycles of 30 s at 94°C, 30 s at 53°C, 1 min at 72°C; 2 min at 72°C. Amplification products were resolved on a 1.8% agarose gel using the 100 bp ladder for markers (Amersham, GE Lifesciences). Bands of 615 and 591 pb for pepsinogen and proton pump were obtained, respectively, purified using GFX PCR DNA and Gel Band Purification kits (Amersham, GE Lifesciences) and ligated into the pCR™4-TOPO® vector using TOPO®TA Cloning® kit for sequencing (Invitrogen, Life Technologies). Recombinant plasmids were transformed into One Shot® Top 10 chemically competent *Escherichia coli* (Invitrogen, Life Technologies). Colonies containing the insert were screened on agarose gels and sequenced using an ABI PRISM® 3100 Genetic Analyzer and the BigDye™ Terminator Cycle Sequencing Ready Reaction Kit (Applied Biosystems, Life Technologies). Sequences were analysed using BlastX (www.ncbi.nlm.nih.gov/BLAST) and amino acid alignments were performed using *DNAstar* (DNAstar Inc, Madison, WI, USA). Fragments were partial cDNA sequences from pepsinogen and proton pump for *D. sargus* and they were used for a gastrointestinal tract cDNA library screening previously constructed and available in our laboratory.

**Table 1:** Oligonucleotides designed for real time RT-PCR.

Name	Sequence (5′→3′)	Position	Amplified fragment (bp)
dsβACT-F	TCTTCCAGCCATCCTTCCTCG	873	108
dsβACT-R	TGTTGGCATACAGGTCCTTACGG	980	
dsHKA-F	TGGTGGTCAGCTGAAAGAGA	535	136
dsHKA-R	GATAGAGCCCAGACGCTGAC	670	
dsPEP-F	CTCAGCAGTCCTCCACCTTC	368	143
dsPEP-R	CTGGCTGATTCCAAACACCT	510	

For library screening, approximately 2.5 10^5^ pfu from the GIT library were plated on each of 2 NZY agar 240×240 mm plates (Nunc), and transferred onto 2 Hybond-N Nylon membranes (GE LifeSciences). Blots were prehybridized for 1 h at 42°C in prehybridization solution (50% formamide, 6× SSPE, 0.5% SDS, 5× Denhart's solution, 1 mg/mL yeast RNA type III). For hybridization, 25 ng of partial cDNA was radiolabeled using the RadPrime DNA Labeling System (Invitrogen, LifeTechnologies) and [α^32^P]dCTP (PerkinElmer), and allowed to hybridize with the Nylon membranes overnight at 42°C. Blots were washed twice in 2×SSC-0.1% SDS for 30 min each at room temperature, twice in 1×SSC-0.1% SDS for 30 min at 42°C, and twice in 0.5× SSC-0.1% SDS for 30 min at 65°C. Membranes were exposed to autoradiography film (Amersham, GE Lifesciences) for two days with intensifying screens at −80°C. Positive plaques were isolated and subjected to further two rounds of hybridization/isolation. After the third round of the screening, 3 plaques were isolated for each and excised to plasmids by in vivo excision using *E. coli* XL1-Blue MRF' and SOLR strains (Stratagene, Agilent Technologies). Excised pBluescript SK(–) plasmids (Stratagene, Agilent Technologies) were double digested by *Eco*RI and *Xho*I (Takara) and the products were revealed in a 1% agarose gel. The clones were full sequenced using an ABI PRISM® 3100 Genetic Analyzer and the BigDye™ Terminator Cycle Sequencing Ready Reaction Kit (Applied Biosystems, Life Technologies).

With this strategy a full-length cDNA clone for pepsinogen from *D. sargus* was obtained, whereas for proton pump the cDNA was partial, lacking the 5′ unstranslated region and a good portion of the 5′ open reading frame. Sequences were sent to GenBank with accession numbers EU163285 and EU274469 respectively.

### Phylogenetic analysis

The evolutionary histories for both pepsinogen and proton pump from *D. sargus* were inferred using the Neighbor-Joining method [37]. The bootstrap consensus tree inferred from 1000 replicates [38] was taken to represent the evolutionary history of the taxa analyzed. The evolutionary distances were computed using the Poisson correction method [39] and were in the units of the number of amino acid substitutions per site. All positions containing gaps and missing data were eliminated from the dataset. Phylogenetic analyses were conducted in MEGA4 [40].

### Q-RT-PCR assays

Tissue samples were preserved in RNA*later* (Ambion) for subsequent total RNA extractions using the RNeasy midi kit and the on-column RNase-free DNase digestion for removal of the genomic DNA contamination (Qiagen). RNA quantity was measured spectrophotometrically at 260 nm with a BioPhotometer Plus (Eppendorf). cDNA synthesis and real time amplification was carried out with the QuantiTect SYBR Green RT PCR kit (Qiagen) and a thermal cycler Mastercycler®ep Realplex^2^ (Eppendorf) with Realplex software (Eppendorf). Q-RT-PCR primers used –shown in Table 1– were designed using the software primer3 (http://frodo.wi.mit.edu/primer3/). β-actin was used as the internal reference gene (GenBank accession number JN210581). Relative gene quantification was performed using the ΔΔC_T_ method [41]. PCR conditions for one step Q-RT-PCR were: Reverse transcription at 50°C for 30 min; an initial PCR activation step at 95°C for 15 min; 40 cycles of 15 s at 94°C, 30 s at 56°C, 30 s at 72°C; and a final cycle of 15 s at 95°C, 30 s at 55°C and 20 min increasing the temperature till 95°C for 30 s to check for primer-dimer artefacts. Controls were performed using single primers (to check for single primers artefacts) and both primers without template (to check for external contamination).

### Statistics

All pH measurements and samples for enzyme activity and gene expression were taken at least in triplicate during different non-consecutive days to prevent potential effect of sampling stress on feeding behaviour. The results are given as mean ± SD. Differences in gastric pH and enzymatic activities measured as a function of time were examined by one-way analysis of variance (ANOVA), followed by Student-Newman-Keuls (SNK) multiple range test. Enzyme activities data were log-transformed prior to analysis to fit homoscedasticity.

## Results

### Phylogenetic characterization of cloned H^+^/K^+^-ATPase and pepsinogen

**Figure 1:**
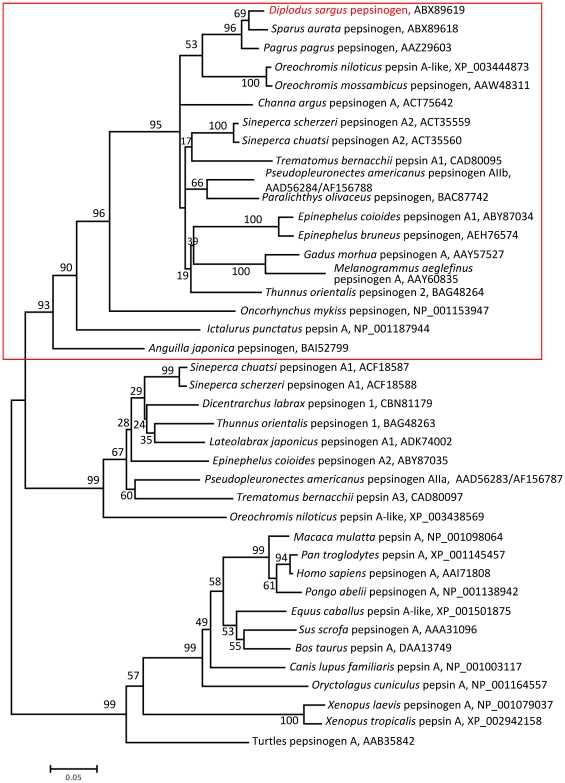
Evolutionary relationships of 40 taxa for pepsinogen precursors. The percentage of replicate trees in which the associated taxa clustered together in the bootstrap test is shown next to the branches. GenBank or NCBI Reference Sequence accession number appears to the right of each taxon.

**Figure 2:**
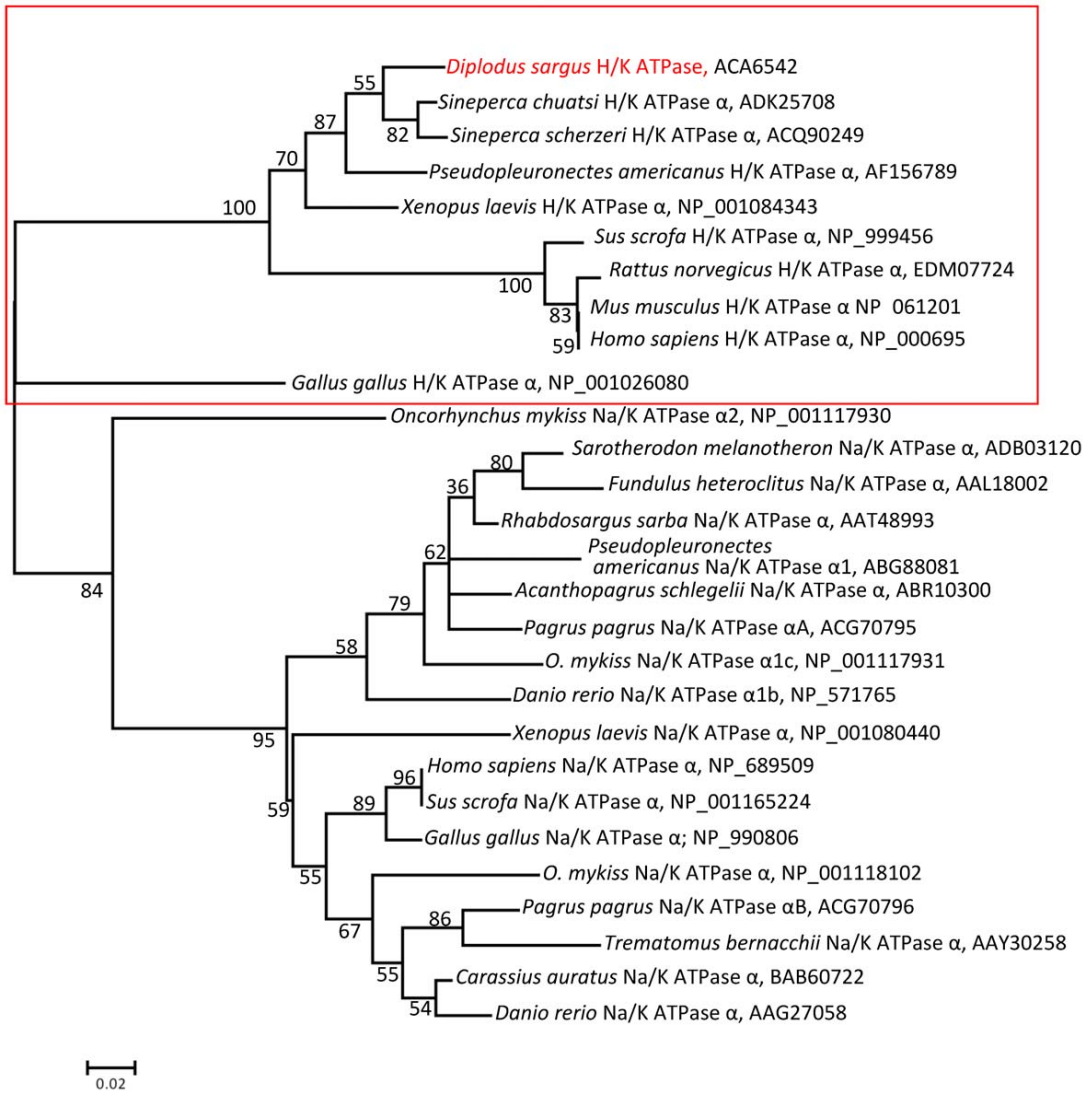
Evolutionary relationships of 28 taxa for proton/potassium and sodium/potassium pumps. The percentage of replicate trees in which the associated taxa clustered together in the bootstrap test is shown next to the branches. GenBank or NCBI Reference Sequence accession number appears to the right of each taxon.

The nucleotide sequences obtained in this study have been deposited in the GenBank under the accession numbers EU163285 for pepsinogen and EU274469 for H+/K+-ATPase. We have constructed the respective phylogenetic trees in order to examine the evolutionary histories of white seabream proton pump and pepsinogen in relation to related genes in other teleosts and vertebrate groups (Figs. 1 and 2). Pepsinogen was aligned with other teleosts, while other vertebrates like mammals and amphibians were aligned in a distinct group (Fig. 1). The identities were 96/99% (in 375 amino acids) with *Sparus aurata*, or 95/98% (in 205 aa) with *Pagrus pagrus*, lower with other fish species depending on the pepsinogen isoform (63–88/77–94%), and the lowest with other vertebrates, like 53/72% (in 369 aa) with *Xenopus laevis*, or 51/70% (in 387 amino acids) with *Homo sapiens*. White seabream H^+^/K^+^-ATPase gen showed 99/99% identity and similarity, respectively (in 536 amino acids), with *Siniperca chuatsi*, 98/99% (in 534 amino acids) with *S. scherzeri*, 96/99% (in 534 amino acids) with *O. niloticus*, and 96/98% (in 513 amino acidos) with *Pseudopleuronectes americanus*. This identity was lower with other vertebrates, like 90/95% (in 534 amino acids) with *X. laevis*, 87/94% (in 534 amino acids) with *H. sapiens*, or 68/82% (in 534 amino acids) in *Gallus gallus*. The identity was even lower to Na^+^/K^+^-ATPases (Fig. 2)

### Digestive response

**Figure 3:**
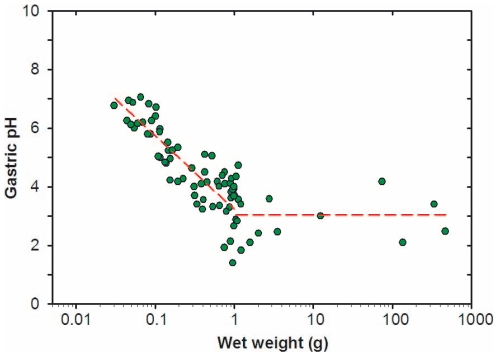
Changes in gastric pH as a function of the wet weight in fed larvae, juveniles and adults of *Diplodus sargus*. Lines indicate the regression of pH in relation to wet weight (W) in fed animals.

Changes in stomach pH with development are shown in Fig. 3. A progressive decrease was observed from values measured in 30 mg wet weight larvae to those in 1 g fish (regression line, pH = 3.24621−2.4482 · Log (wet weight), r = 0.860, p<0.01). Above this weight and up to the adult size, the gastric pH was maintained between 2 and 4.

**Figure 4:**
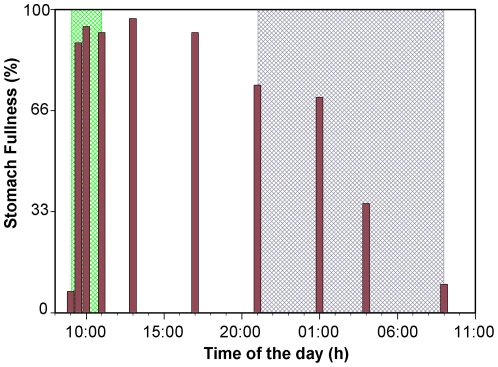
Relative stomach fullness during the 24 h cycle in juveniles of *Diplodus sargu*s. Green area: feeding period. Grey area: dark period.

**Figure 5:**
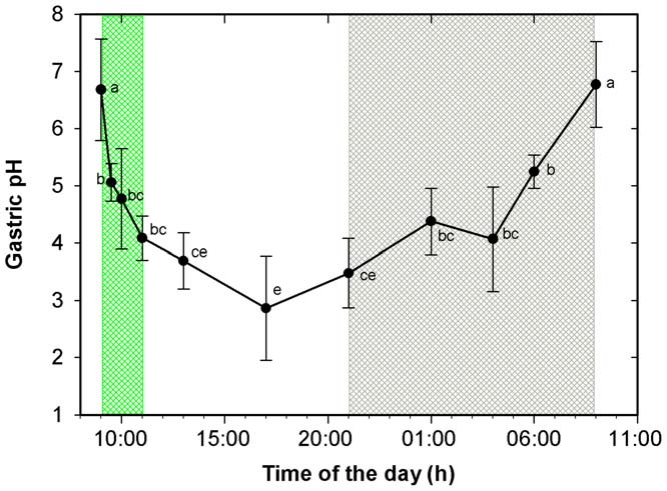
Gastric pH (mean ± SD) during the 24 h cycle in juveniles of *Diplodus sargus*. Same letter indicates no significant difference (*P*>0.05). Green area: feeding period. Grey area: dark period.

In the postprandial response experiment it was observed that fish showed a plenty stomach immediately after the food supply, being food transit towards the intestine initiated 12 hours later and the stomach became empty after 24 hours (Fig. 4). Gastric pH in the first sampling was close to 7 and decreased quickly after food supply, reaching values below 4 four hours later. The minimum gastric pH was recorded at 17:00 h, 8 h after the food supply, although values measured in samples obtained between 12:00 h and 21:00 h were not significantly different (P>0.05). From this time onwards, gastric pH increased steadily to reach the initial neutral values just before the next food supply (Fig. 5).

**Figure 6:**
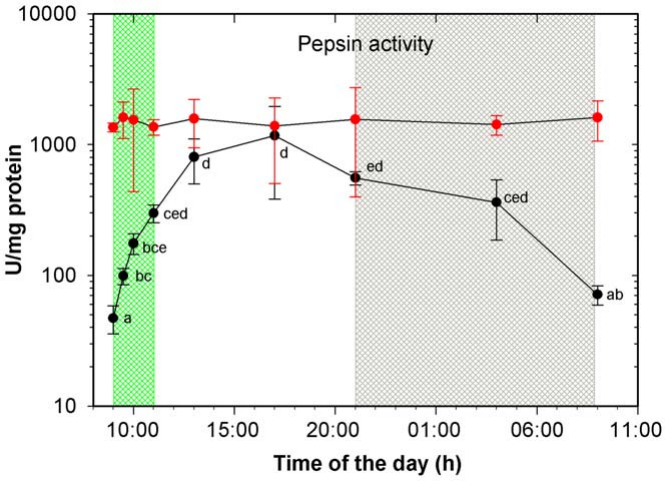
Pepsin activity (mean ± SD) during the 24 h cycle in juveniles of *Diplodus sargus*. Red line indicates the analyses done at pH 2. Black line indicates the analyses done at the actual luminal pH. Same letter indicates no significant difference (*P*>0.05). Green area: feeding period. Grey area: dark period.

Values of pepsin activity measured at pH 2 showed quite constant values during the whole 24 h period (Fig. 6). Nevertheless, when the pH of the buffer used in the enzyme analysis was adjusted to that actually measured in the stomach, the daily pattern of pepsin activity showed important changes. The average values increased progressively from 50 U/mg protein just after the food supply up to 1168 U/mg protein at the moment of the lowest registered pH in the stomach. Then, the activity decreased progressively to initial values (Fig. 6).

**Figure 7:**
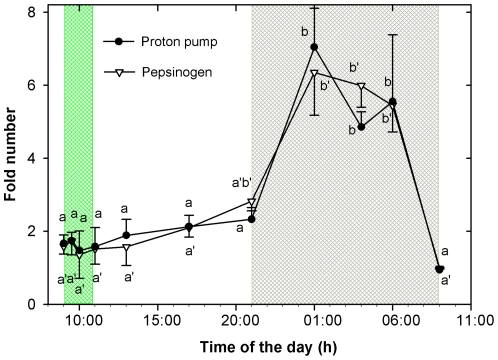
Gene expression of pepsinogen and H^+^/K^+^-ATPase mRNA during the 24 h cycle in juveniles of *Diplodus sargus*. Same letter indicates no significant difference (*P*>0.05); a and b: proton pump; a′ and b′: pepsinogen. Green area: feeding period. Grey area: dark period.

Gene expression of pepsinogen and proton pump showed almost parallel patterns (Fig. 7). A slight non-significant increase was observed during the light hours from 9:00 h to 21:00 h. After the start of the dark period, the expression showed a sharp increase of several folds over the basal values and it was maintained high during almost the whole period although a clear reduction was measured in fish sampled just before the morning meal. Maximum expression values ranged between 5 to 6 fold and were determined between 1:00 h and 6:00 h.

## Discussion

Different pepsinogen groups (A, B, C-progastricsin, F and Y-prochymosin) and isoforms differing in structure, protein cleavage position and optimum functional pH have been described in vertebrates [42]. In fish, from one to four different gastric pepsinogen isoforms have been described in the different species [43] and their nomenclature varies among researchers. Some studies classify the pepsinogen as pepsinogens A, B, and C, while in other pepsinogens are named from I to IV. In the present study, we have cloned a full-length cDNA encoding a pepsinogen isoform showing a high homology with pepsinogen A of teleosts, and the highest identity with that in other sparids. In the case of proton pump, there are fewer genes publicly available for comparison. The highest identity was obtained with other teleost fish, but it was also high with other vertebrates like *X. laevis* and mammals. Such homology among vertebrates belonging to diverse taxonomic groups should indicate that it is a highly conserved sequence and, therefore, plays a major role in the survival of organisms. Interestingly, the Na^+^/K^+^-ATPase (sodium/potassium-activated adenosine triphosphatase) genes related with uptake of ions (Na^+^, Cl^−^, Ca^2+^) from the surrounding water in the branchial epithelium, were clustered close to H^+^/K^+^-ATPase genes indicative of the common origin.

In marine fish with altricial development, the appearance of a functional stomach takes place usually several weeks after the onset of feeding. Gastric glands are developed progressively during a variable period in different species [44], [45]. In the white seabream the first gastric glands appear by day 20 after hatching at 20°C of temperature [46] and pepsin activity rises notably from day 25 after hatching [47].

The increase in acidification capacity with development observed in the present study has been also reported in other species [48], [49], [50], [17]. Interestingly the complete capacity was attained when juveniles reached around 1 g wet weight (40–45 mm total length) irrespective of the age, this being in agreement to results obtained with another species of the family Sparidae with similar feeding habits; the gilthead seabream *S. aurata* [17]. At this size, most of the juvenile features have been attained and the fish move definitively to a benthic environment, showing a notable change in their prey spectrum and feeding habits.

The secretion of gastric acid, either produced to maintain basal levels or triggered by food intake, is regulated by complex mechanisms including neural and endocrine control through neurotransmitters and hormones [51]. Many details of this basic regulation in teleost fish are still unknown and, in addition, such secretion may follow different functional patterns. The present study was designed as a preliminary approach to assess these mechanisms using the simplest scenario; the postprandial response of some key features of the acidic digestion produced after a single meal in a regular day/night cycle.

Our results confirm that, as it has been observed in other teleosts, the white seabream maintains a neutral pH in the stomach lumen during fasting, being acidification only stimulated by the ingestion of food. This results in an immediate and sharp decrease of gastric pH in the area close to the stomach mucosa, similarly to what reported for the gilthead seabream [16], [17], [18]. In the present study, the minimum pH values determined during the 8 hour period following food intake ranged between 3.0 and 3.7. These values are within the upper range or even slightly higher than the optimum pH for pepsin activity in different sparids [29], [31], [33], [52]. Transformation of pepsinogen into pepsin requires a pH decrease below 4.0. Most data of pepsin activity reported in the literature for different fish species were obtained with assays performed at pH 2 and did not consider the actual pH in the stomach of the species. But gastric pH in young fish may be far above that optimum used in the standard laboratory and this may lead to an over-estimation of the real activity, since an important fraction of the enzyme may be still present as inactive zymogen at a higher pH. In the present study, the total amount of pepsin in the different samples showed no changes with time when assays were performed at pH 2, while a fraction of such activity, ranging from 3% to 80% was measured when assays were performed at the pH measured in the stomach in each sample. Hence, the amount of active pepsin should be linked to an effective pH decrease, which is produced only when enough amount of hydrochloric acid is secreted after food ingestion.

Changes in pepsinogen mRNA expression in fish has been analyzed mainly in the late larval period during the transition to juvenile in *Paralichtys olivaceus* [53], *Hippoglossus hippoglossus* [54]; *Melanogrammus aeglefinus*, *Gadus morhua* [55], *Oreochromis mossambicus* [56] and *Epinephelus coioides* [57]. The gene expression of both pepsinogen and proton pump expression has been only analysed in postlarvae of *P. americanus* [36], *P. pagrus* [5] and *Pelteobagrus fulvidraco* [58]. In juveniles and adults, time curse or postprandial experimental studies at molecular level are practically absent. Only the effect of dietary acidification on proton pump gene expression in *Oncorhynchus mykiss* [12] and the effect of fasting and refeeding on pepsinogen C gene expression in *Dicentrarchus labrax* [59] have been reported.

In contrast to what described for gastric pH and pepsin activity, daily changes in mRNA expression of pepsinogen and proton pump were not particularly related to the moment of food supply, but modulated by the circadian cycle. The results clearly evidenced how the mRNA transcripts, which showed low levels during the diurnal and feeding period, were overexpressed during the night-time, this meaning that the transcriptional effort is done during the resting hours. This contrast to the almost stable amount of pepsinogen/pepsin measured at any moment during the experiment. This suggests that translation of pepsinogen mRNA to protein may be constant in order to maintain a stable amount of zymogene. Such assumption implies that the activation of the proton pump is the key process regulating the acidic digestion.

The regular feeding pattern in the present study allowed fish to establish a digestive response of anticipation to the moment of food supply. Montoya et al. [21] found that the daily pattern of gastric pH in adults of *S. aurata* is modified when changed from regular to random feeding. However, as described in the present study, the pepsin activity determined a pH 2 did not show a daily rhythm and was not affected by the feeding protocol. As far as we know, there are no reports of postprandial mRNA expression patterns of pepsinogen and proton pump in animals. Nevertheless, Terova et al. [59] found that expression of the pepsinogen C gene was modulated by the feeding status in adults of *D. labrax*. Such expression was stable during weeks when the fish were regularly fed but was downregulated under prolonged starvation and upregulated when the fish were re-fed. All these results suggest that mechanisms controlling the acid digestion are adaptable to potential changes in food availability.

The mRNA expression of both the pepsinogen and the proton pump exhibited a quite similar pattern. This could be due to the fact that both are produced in the same oxynticopentic cells of the gastric glands, and may share the same regulation mechanisms. Gawlicka et al. [3], using *in situ* hybridization technique, also found that pepsinogen and proton pump were expressed simultaneously in the gastric glands of *P. americanus*. It has been argued that this simultaneous secretion could be a way to provide a rapid conversion of the pepsinogen to the active pepsin [60]. Nevertheless, this is not a general mechanism since gastric acid and pepsinogen secretion show different sensitivities to the stimulatory agents and may be separately released from the oxynticopeptic cells [61] as may also be deduced from our study.

In conclusion, our study describes for the first time in vertebrates an integral postprandial response of the acid digestion including changes in two key enzymes at both biochemical and molecular levels. We provide evidence that the white seabream, as many other teleosts, tend to maintain a neutral gastric pH during fasting which drops quickly after food ingestion. We also provide evidence for the first time of the accumulation of mRNA of digestive gastric enzymes, pepsinogen and proton pump, taking place during the nocturnal and resting hours. Our findings suggest that while the translation of pepsinogen is continuous, the translation and/or activation of H+/K+-ATPase depends on food ingestion. The relevance of this study is that describes a basic response pattern useful for the understanding of the regulation mechanisms of acid digestion in fish and other vertebrates under different feeding situations.
